# Three individuals, three stories, three burials from medieval Trondheim, Norway

**DOI:** 10.1371/journal.pone.0180277

**Published:** 2017-07-03

**Authors:** Stian Suppersberger Hamre, Geir Atle Ersland, Valérie Daux, Walther Parson, Caroline Wilkinson

**Affiliations:** 1 Department of Archaeology, History, Cultural Studies and Religion, University og Bergen, Bergen, Norway; 2 Laboratoire des Sciences du Climat et de l’Environnement, UMR CNRS/CEA/UVSQ/IPSL, Gif-sur-Yvette, France; 3 Institute of Legal Medicine, Medical University of Innsbruck, Innsbruck, Austria; 4 Face Lab, Liverpool Science Park IC1, Mount Pleasant, Liverpool, England; University at Buffalo - The State University of New York, UNITED STATES

## Abstract

This article presents the life stories of three individuals who lived in Trondheim, Norway, during the 13^th^ century. Based on skeletal examinations, facial reconstructions, genetic analyses, and stable oxygen isotope analyses, the birthplace, mobility, ancestry, pathology, and physical appearance of these people are presented. The stories are discussed within the relevant historical context. These three people would have been ordinary citizens, without any privileges out of the ordinary, which makes them quite rare in the academic literature. Through the study of individuals one gets a unique look into the Norwegian medieval society.

## 1. Introduction

This article will tell the stories of three individuals who ended their lives in medieval Trondheim, Norway. They may have been contemporaries, they may have known each other, or they may have been completely oblivious to each other’s existence. What is for certain is that these three people were buried in the same graveyard in Trondheim [called Nidaros at the time but will only be referred to as Trondheim throughout this paper) during the period between 1175 and 1275 [1]. However, according to analyses of their skeletal remains, their life stories were rather different. Not only will this article present the stories of three people who died around 800 years ago, but these individuals’ stories will also provide new information about medieval society in Trondheim as well as society outside this town and further afield.

During a wider investigation of the medieval and post-medieval population of Trondheim, three individuals were chosen for more analysis. Personal stories of ordinary individuals are quite rare in the academic literature(e.g. [2]). Kings, queens, and other members of the privileged few have, of course, been discussed in detail on a personal level, like king Sverre (died 1202) and his descendants who ruled Norway until the early 14th century (e.g.[3, 4]). Such elite members of society are known from the saga literature and documented through deeds. This also applies to prominent members of both the lay and secular aristocracy, but the rest of the population has been more difficult to access on this level and has largely been ignored. In the following, the authors will attempt to rectify this situation by presenting three people who walked the streets of medieval Trondheim without any privileges out of the ordinary.

The life stories of these individuals are, to varying degrees, remarkable, but they are representative of the population of medieval Trondheim and exhibit diversity regarding birth place, ancestry, and life history. The heterogeneity of the population becomes visible through the study of individuals in a different manner than larger population studies manage (e.g. [5, 6]). It has been possible to gather information about these people which is of such a standard that it is possible to undertake qualified discussions about their lives above and beyond the facts alone, without having to resort to pure speculation.

## 2. Place of burial

The remains of these people were recovered from the Library site in Trondheim which was excavated during 1984 and 1985 [7]. There is some uncertainty with regard to which church the Library site graveyard belonged to. It was believed that the church at this site was the St. Olav’s church, but this has been challenged [8, 9] and at present there is no agreement as to the name of this church. What is known, however, is that this church dates to the early 12th century and that it became part of a Franciscan monastery in the late 13th century [8]. From this, it can be assumed that this was not a monastic church before this point, and that at the time the individuals in question were buried it is likely to have been a parish church. The Library site church was centrally located within the densely-built area of medieval Trondheim and was one of 14 contemporary churches within the borders of the town’s jurisdiction.

The Library site graveyard has, archaeologically, been divided into three burial phases [1] which have each been dated, making it possible to determine the time of burial for these people with reasonable accuracy. All three belong to burial phase B which dates to between 1175 and 1275 [1].

## 3. Methodology

All three individuals have been subjected to stable oxygen isotope analysis (δ^18^O) of the enamel on the first and the third molars as well as ancient DNA (aDNA) analysis. The skeletal remains of these people have also been examined anthropologically to determine sex, to estimate age at death and living stature, and to establish their accessible history of disease and trauma. Radiographs were taken of the tibiae to look for Harris lines to assess possible episodes of stunted growth (for a discussion of Harris lines, see: e.g. [10–13]), and the cranium of one of the individuals was also radiographed due to suspected porotic hyperostosis (for discussion of porotic hyperostosis, see: e.g. [11, 14, 15]).

The study was approved by “The national committee for research ethics on human remains”: case 2012/223 (https://www.etikkom.no/en/our-work/about-us/the-national-committee-for-research-ethics-on-human-remains)

### 3.1. Anthropological examination

The skeletons had already been examined by Trevor Anderson [7] and were later re-examined by the first author [16] and, therefore, yet another estimation of age at death and living stature, and determination of sex, was deemed unnecessary for the purposes of this study. A closer investigation of pathology and trauma was, however, undertaken (by the first author) to create a best possible picture of these peoples’ histories of disease and injury.

### 3.2. Radiographic analysis

The radiographs were taken with Industrial X-RAY Equipment Eresco 42 MF3 with Digital Control “ERESCO Control” from GE Inspection Technologies. Standard film focus distance (between the X-ray tube and the film) is 70 cm. For the radiographs of the tibiae an AGFA Structurix D4 Pb (48cmX10cm) film was used. The bone was placed on the film and its identity number was marked on the film with numbers made of lead. The voltage employed was 65 kV, the tube currency 4,5 mA and the exposure time 3 minutes. For the cranial radiographs, an AGFA Structurix D5 Pb (24cmX10cm) film was used. Here the voltage was 60 kV, the currency 4,5 mA and the exposure time 9 minutes.

### 3.3. δ^18^O analysis

The oxygen composition in enamel apatite carbonates (δ^18^O_C_) from the first (M1) and the third (M3) molars has been analysed for the three individuals. The analyses were carried out by GNS Science (Wellington, New Zealand) and the results were reported on the V-SMOW scale. The δ^18^O_C_ data was then converted to δ^18^O_w_ (oxygen composition in water/precipitation) by using equation 6 from Daux et al. [17] modified by Chenery et al. [18]. Application of this conversion leads to a model uncertainty of ±1.0‰ (2 σ) according to Chenery et al. [18].

Breastfeeding affects the oxygen composition in the body [19–21] and, consequently, the oxygen composition in the enamel of M1, as the crown of this tooth forms during the period when children are normally breastfed. The timespan for breastfeeding would have been similar during the Middle Ages [22]. Taking this into account, the δ^18^O_C_ of the M1 may be 0.2‰ to 1.0‰ higher than it would be if the baby had the same diet as a weaned child [5]. For determination of where individuals lived, the model uncertainty of ±1‰ (conversion from δ^18^O_C_ to δ^18^O_w_) and the breastfeeding effect (diminution of 0.2‰ to 1.0‰ must be applied to the δ^18^O_C_ of M1) are taken into account.

Another variable influencing the isotope composition in precipitation is temperature. Average yearly temperatures fluctuate from one year to another and, thus, influence the isotopic composition of precipitation accordingly. Applying the formula developed by Dansgaard [23] for traducing δ^18^O_w_ from temperature, a 1°C increase equals an increase of δ^18^O_w_ by 0.7‰. Thus, it is important to assess the average temperature at the time the examined teeth developed. For the purpose of the three individuals discussed in this paper, the exact year of birth is not known but they were most likely born during the first half of the 13th or possibly towards the end of the 12th century. The temperature reconstructions of the growing season temperature in west-central and central Scandinavia [24–26] show that, at least in summer, the temperature from the late 12th century to the early 13th century was not much different from the temperature in the 20th century. To our knowledge, there is no available reconstruction of temperature in Scandinavia for the other seasons. As neither the dates of birth of the individuals, nor the temperatures from autumn to spring in Trondheim are precisely known, no specific corrections due to possible temperature differences between the lifetimes of the individuals and the present time were applied.

The δ^18^O_w_ for drinking water in Trondheim has been determined by Hamre and Daux [5] to -8.7‰ and comparative δ^18^O_w_ data for the rest of Europe has been gathered from GNIP (Global Network of Isotopes in Precipitation) precipitation collection stations and various publications.

### 3.4. aDNA analysis

Ancient DNA analyses on the human remains comprised three main laboratory processes: DNA extraction, DNA typing, and results interpretation, all performed at the Institute of Legal Medicine (GMI), Medical University of Innsbruck, Austria. The analyses were performed in a forensic molecular genetic laboratory designed and optimised to meet the required high quality. After photo documentation of the individual specimens and weighing of the received samples, work lists were generated to pursue molecular genetic analyses.

#### 3.4.1. DNA extraction

In order to avoid the possibility that DNA (modern) located on the surface of the samples could interfere with the analysis, the remains were thoroughly cleaned. The surfaces of the fragments were mechanically cleaned with sterile scalpel blades. The samples were then washed (NaClO, purified H_2_O, absolute EtOH), dried, and ground using a horizontally oscillating ballmill. Bone powder was dissolved in a hybridisation oven, the DNA extracted with a modified silica-column-based Spin Filter (SF) method [27, 28].

#### 3.4.2. DNA typing

The total amount of nuclear (n)DNA and mitochondrial (mt)DNA present in the purified DNA extract was determined by means of a modified real-time (rt)PCR DNA quantification approach [29, 30]. The targeted human-specific sequences were AluYb8(71bp) and mt143bp(143bp). A 7500 Real-Time PCR System and 7500 Software v2.0.6 were used in both quantification assays. Molecular genetic sexing was performed using redundant gonosomal information (Amelogenin, SRY-gene, X-STRs) according to the GenderPlex protocol detailed in Esteve Codina et al. [31]. For the data analysis GeneMarker HIDv1.70 Software was used. Y-chromosomal DNA in the male sample was typed with the PowerPlex Y23 System and the Yfiler Plus PCR amplification kit. Raw data were analysed using GeneMapper ID-X Client Software. The consensus Y-STR haplotype was assigned to the respective Y-chromosomal haplogroups (hg) [32, 33]. The mtDNA control region (CR) was sequenced for all samples using the mini-multiplex assay designed by Berger and Parson [34]. All sequences were run on a 3500xL Genetic Analyzer. Raw sequence data were analysed and aligned using Sequencher vs.5.1.

### 3.5. Facial reconstruction

The skulls were assessed and facial depictions produced by Face Lab at Liverpool John Moores University. The depiction of faces of the dead can be a useful tool for promoting recognition leading to forensic identification. Post-mortem facial depiction (otherwise known as reconstruction or approximation) is described as the interpretation of human remains in order to suggest the living appearance of an individual [35]. It is well established that the craniofacial complex is a functional matrix and the relationship between the bone and soft tissues is reciprocal and responsive [36, 37]. Therefore, the principles of craniofacial analysis and identification are based on the theory that the shape of the adult skull is related to the internal and external soft tissues of the face and head, as much as the face and head are related to the skull. There are a number of anatomical and anthropometrical standards that are employed during the facial depiction process, and these rely on the interpretation or measurement of skeletal structure to estimate soft tissue features. In this way the orbits are interpreted to predict the eyes, the nasal aperture is measured to predict the nose, the teeth are analysed to predict the mouth and the mastoid processes are assessed to predict the neck and ears. These standards [37–39] have been established from studies relating to human dissection [40–46], clinical imaging (CT, MRI and X-rays) of living subjects [47–50], palpation studies [51, 52] and anthropometry [37, 53].

In addition, tissue depth data at anatomical points across the skull are utilised to act as guidelines for the amount of soft tissue over the skull as related to sex, age and ethnic group. Throughout the 20^th^ and 21^st^ centuries clinical imaging methods have been utilised to measure living subjects, starting with planar x-rays and eventually advanced 3D imaging, such as computed tomography, Magnetic Resonance Imaging, ultrasound, low dose cone-beam computed tomography and holographic topometry. There are many sets of data available from different in vivo ethnic groups across the world for use in craniofacial analysis. There are data for adults from a variety of ethnic groups in Germany, Belgium, the former Soviet Union, Slovakia, Japan, South Korea, The Netherlands, USA, Egypt, Chile, Turkey, India and South Africa. There are datasets for children from the USA, the UK, Japan and the Philippines. Collations of these datasets can be found in some publications [38, 54] and at some websites (www.craniofacialidentification.com; www.craniofacial-id.com).

The skulls were scanned using Artec Space Spider, which is a high-resolution 3D scanner based on blue light technology. The scanner’s ability to render complex geometry, sharp edges and thin ribs means it is an ideal industrial 3D scanner for high resolution capturing of objects followed by the export of the final 3D model as an obj. file. The scan data was translated into 3D digital models of the skulls in order to produce facial depictions in our 3D computer system. The system employs Geomagic Touch X hardware and Freeform Modelling Plus software. This system has haptic feedback and allows the practitioner to feel the surface of the model that can be seen on the monitor. A database was accessed for the depiction process containing pre-modelled muscles and anatomical structures created for use in forensic reconstruction.

This facial depiction system has been tested using the skulls of living subjects [55, 56] and these studies suggest that it is possible to create a face that is recognisable, with 67% of the surface of the face showing less than 2mm of error. Face Lab also utilised high quality photographs of the skulls and some details of the archaeology/anthropology report relating to disease and trauma.

## 4. Three individuals, three stories

### 4.1. Why were these three chosen?

The reason for selecting these three individuals was to illustrate diversity in medieval Trondheim, but the selections had to be made within some limitations and according to certain criteria. Facial reconstructions were going to be made from the remains of these individuals, for use in a museum exhibition at Bryggens Museum in Bergen, Norway. Thus, the first criterion was that the skull had to be sufficiently complete. This excluded about half of the 41 medieval skeletons which had been examined and analysed and were, therefore, potential candidates for selection. After that, isotope results were the deciding factor for choosing SK152, the mtDNA profile was important for including SK196, while the last individual, SK259, was chosen more at random with the only deciding criterion being that he was male. Other information about pathology and trauma was added later and were not factors in selection. Thus, SK152 was chosen due to her oxygen isotope values showing that she had been born a long distance away from Trondheim, as well as having travelled a significant distance during her childhood years. SK196 was chosen because she belonged to a genetic haplogroup suggesting her ancestry was not local to Trondheim or Norway, and this case is also a good example of how genetic and isotopic evidence can work together to create a clearer picture of life history.

#### 4.1.1. Representativity

These three individuals were not chosen at random and can, by no means, be said to represent the whole spectrum of the population in medieval Trondheim; three individuals never can. However, they have been chosen to represent some of the variety within the population while at the same time not being outliers in society. Their place of burial provides a clue as to their position in society. The ecclesiastical parts of the provincial laws (the Frostating, Gulating, Borgarting and Edsivating laws), which were in operation at the time these people died, give different regulations about burials. The most important in this context is that the graveyards should be socially stratified, with the upper layers of society being buried close to the church and the people pertaining to the opposite end of the social hierarchy being buried close to the graveyard fence (For a discussion of the ecclesiastical legislation, see [16, 57, 58]). The socially stratified graveyard is also supported osteoarchaeologically [16]. The three individuals discussed in this paper were all buried centrally in the graveyard and one can, therefore, assume that they were not among the privileged few in the population and did not belong to the lowest strata of society. Thus, socially they are likely to represent the largest, “normal”, part of the population.

Their ages at death ranges from early twenties for SK152 to early thirties for SK259. This may seem young, but would have been more like the norm than the exception in the Middle-Ages. A recent study from medieval England shows that the great majority of the adult population died before the age of 40 and nearly half before the age of 30 [59]. This corresponds very well with data from medieval Norway, including the Library site [16].

What about their places of origin and mobility patterns, can that be said to be representative of the population in medieval Trondheim? A recent study of mobility in the population in Trondheim [5] showed that at least 40% of the medieval population were born outside of the town and as much as 36% of the medieval population moved to, or towards, Trondheim during their childhood years. Most of these were shown to have had their origin in areas to the north or east of Trondheim, but the study also suggest that a smaller group of people is likely to have come from central or southern Europe and/or possibly the British Isles. Thus, the three individuals, discussed below, would not have been outliers in medieval Trondheim, but rather representatives of an urban heterogeneous population.

### 4.2. The girl who travelled far: SK 152 (Fig 1)

**Fig 1:**
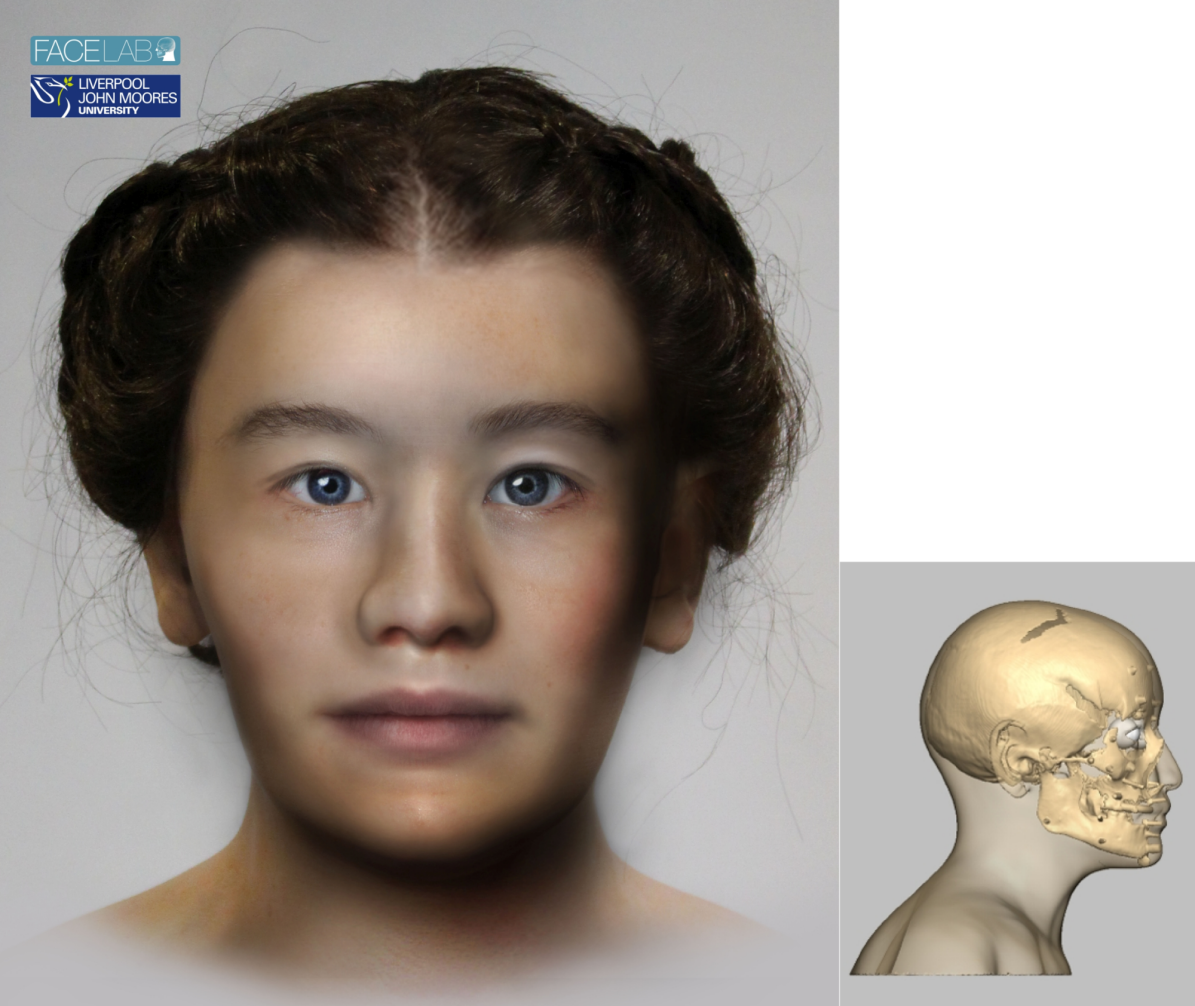
The left picture shows the facial depiction of SK152 while the right picture shows the profile view of the textured skull of the same woman.

#### 4.2.1. Sex, age at death, and stature

The sex of this person was determined morphologically by Trevor Anderson to be female [7], a conclusion later confirmed by the first author [16]. Recently, genetic sexing (Genderplex analysis) has confirmed beyond doubt that this individual was a woman, and it is also clear that she died when she was in her early twenties [7, 16]. Her living stature has been estimated on the basis of the 401mm maximum length of the left femur, indicating she stood between 150.9cm and 159.9cm tall during her adult life [60].

#### 4.2.2. Geographic origins and movement

The δ^18^O values for the carbonates of the enamel from the first and the third molars are 21.13‰ and 24.72‰ respectively. Hamre and Daux [5] have shown that the δ^18^O_w_ corresponding to the M3 tooth was about -9.33‰ (±1‰), which is compatible with Trondheim. Taking into account the possible effect of breast-feeding effect on the M1 tooth, they have calculated the isotopic gradient the individuals crossed between early childhood and teenage. They have shown that this woman was neither born in, nor near, Trondheim where she ended her life, but in an area where the environmental water was 6‰ to 7.3‰ lighter than in Trondheim, that is with δ^18^O_w_ of about -15 to -16.5‰. The δ^18^O_w_ values of mean precipitation (GNIP database) and surficial waters [61] range between about -11‰ and -14‰ in Scandinavia, except in Naimakka in the county of Norrbotten, northern Sweden (-16.6‰), which would have similar environmental and climatic conditions to the inner regions of the county of Finnmark in Norway and the northwesternmost parts of Finland. Further east, δ^18^O_w_ values at this level of depletion are found in northwestern Russia in the regions of Komi, the Nenets Autonomous Okrug, and even further inland in the southern parts of Krasnoyarsk (GNIP database and [62]). Looking westward, such values can be found on the east coast of Greenland and, looking even further west, the Canadian provinces of Newfoundland and Labrador. These kinds of δ^18^O_w_ values are also found in the southern parts of Ontario and Manitoba (GNIP database and [63]).

A birthplace in Greenland is, however, unlikely as δ^18^O_w_ values as low as these are only found northwards along the east coast where there were no settlements at the time. The contemporary settlements on Greenland were found on the southern tip and the southwestern coast and neither of these areas are compatible with this woman’s M1 values. It is also difficult to argue for a North American origin even though contact with the continent had been made a couple of centuries earlier [64, 65]. There is little evidence for trade or frequent voyages between the Norse world and the American continent at this time and, although it is by no means impossible, it is difficult to build a convincing case for this possibility on the current evidence. Thus, a place of origin for this woman in the inner regions of the northernmost parts of Scandinavia or in northwestern Russia seems by far the most likely.

#### 4.2.3. Ancestry

The mitochondrial (mt)DNA Control Region (CR) haplotype of this woman was determined as 16232T 16235G 263G 309.1C 315.1C 523DEL 524DEL aligned against the corrected version [66] of the first sequenced human mitochondrial DNA [67] and aligned according to standard forensic practice [68, 69]. This haplotype was not found in the largest forensic mtDNA database EMPOP (www.empop.online, v3/R11; [70]) nor in other online resources, suggesting that this haplotype is rare. This CR motif has been observed in hg H4a that is today spread over the European subcontinent. with a centre of gravity identified on the Iberian Peninsula, in particular in northern Spain (www.empop.online, v3/R11). Hg H4a has also been found in the Neolithic Cardium Pottery culture in Portugal and Spain (http://www.eupedia.com/europe/Haplogroup_H_mtDNA.shtml).

#### 4.2.4. Cranial morphology and physical appearance

The skull was assessed morphologically and anthropometrically. The skull suggested a small, gracile female with mild brow ridges, a sloping jaw line, a moderately wide palate, round orbits and an upright profile. The skull was typically female in skeletal characteristics.

The determination of eye morphology is related to the position of the inner and outer canthi and the position of the eyeball in the orbit. Previous research [40, 46] described the inner canthus as determined by the anterior lacrimal crest and the malar tubercle. The eyeball position is determined with the iris touching a tangent across the mid-supraorbital to mid-infra-orbital bone [50] and the eyeball is positioned closer to the roof and the lateral wall of the orbit [46]. The average eyeball diameter is 24 mm, with a 12mm diameter iris and the eyelids hugging the eyeball closely whilst clipping the edge of the iris as they cross the eyeball. With these details determined and sculpture of the correct anatomical structures, the eyes of SK152 were depicted with upward sloping (laterally) eye fissures and normal eyeball protrusion. The strong anterior lacrimal crest and low nasal root suggested that SK152 had epicanthic folds. Epicanthic folds are a feature associated with Mongoloid-type skulls that originate from Asia outside of the Indian sub-continent, Native American or Inuit populations. The eyebrow pattern is related to the shape of the brow and nasal root [52] and the open orbits and low nasal root suggested that SK152 had arched eyebrows.

Research has shown that the nasal aperture at its widest point will be three-fifths of the overall width of the soft nose [41, 71] and the shape of the nasal aperture is directly related to the soft tissue nose. Guidelines for nasal shape prediction have shown a high level of reliability [72] using several measurements and angles along with morphological interpretation. The presence or absence of nasolabial creases is related to the depth of the bone at the canine fossa and the tilt of the base of the nose is related to the direction of the nasal spine [41, 72]. With these details determined and the sculpture of the nasal features (nostrils, alae and columella), the nose of SK152 was depicted as small, with a straight dorsal ridge, a rounded nasal tip, up-turned columella and no visible nasolabial creases.

The morphology of the mouth is an area of the face where there is more reliance on artistic interpretation. Orthodontic and anatomic research shows that the occlusion of the teeth [73–79], the dental pattern [80] and the facial profile [41] will all be related to mouth form. Where the upper teeth are more prominent than the lower teeth, the upper lip will be more prominent than the lower lip and vice versa, and different occlusion patterns will suggest different lip patterns [41]. There are some standards for determination of mouth shape, such as placement of the fissure just above the middle of the maxillary incisor crowns and the mouth corners on radiating lines from the first premolar-canine junction [40]. There is also a positive correlation between the upper lip thickness and maxillary enamel height and lower lip thickness and mandibular enamel height [53] and a set of regression formulae can be utilized for a White European population. However, the exact shape of the vermillion line is difficult to predict with any degree of accuracy and successful reconstructions are demonstrated where the practitioner has modelled the lips ‘in sympathy’ with the rest of the face. The lips of SK152 were determined as moderately thick with up-turned corners, a narrow philtrum and lips of equal prominence. The skull of SK152 also suggested a small chin and angled jaw line.

The ear shape can also be very difficult to determine. The angle of the ear is considered parallel to the jaw line and if the mastoid processes are directed downward, the earlobe will be attached (adherent), whereas if the mastoid processes point forward, the earlobe will be free [41], but otherwise very little information regarding ear shape can be determined reliably. Typically standard ear models will be attached to the reconstruction, which vary in relation to size, prominence and lobe pattern only [38]. For SK152 small, lobed ears were attached. The neck is determined by the position of the mastoid processes where the sternocleidomastoid muscles attach and SK152 showed a small gracile neck.

The next stage of the facial depiction process is the addition of a skin layer over the muscle structure to fill the face out to the level of the tissue depth pegs. This skin layer follows the shape of the muscles below, so that the main determinant of facial morphology is anatomical structure. For SK152 minimum European female tissue depth measurements were used to reflect a 13th century diet and lifestyle.

Finally, the finished head is textured to add skin colour, eye colour, hair colour and style. Here the advice of experts at Bryggens museum in Bergen was considered using typical hairstyles, and the DNA results informed the choice of brown hair and blue eye colour, and the light skin was considered the most likely based on where she was found and her estimated birthplace. This part of the process was carried out in Face Lab using Adobe Photoshop and a 3D stereolithographic copy of the head was also produced for the museum exhibition.

The final head shows a delicate female face with small features and ambiguous ancestry.

#### 4.2.5. Pathology and trauma

There is no visible evidence of trauma or pathology on this skeleton. The only potential point of interest is that she had lost her left mandibular second premolar and the alveolus was closed and well healed. There are also no Harris lines visible on the radiograph of the left tibia and there is no evidence of hypoplastic enamel, which suggest that this person did not suffer any severe episodes of either disease or starvation during growth.

#### 4.2.6. Discussion

When combining the information from the δ^18^O and the mtDNA analyses of this individual, it is possible to perform some qualified speculation regarding this woman’s life history. The isotope result from the first molar tells us that she was born quite far away from Trondheim, in northern regions, while the mtDNA analysis suggests that her maternal ancestry may be found somewhere else again. The haplogroup H4a is mainly found in western and central Europe, but is not common in the geographic regions where this woman could have been born. This suggests that this woman’s maternal ancestors were not native to where she was born. With the haplogroup H4a found principally in central and western Europe and the hot spot being found in northern Spain, one may consider a scenario where the maternal lineage of SK152 can be traced back to one of these more southern or central areas of Europe. Regarding her paternal ancestry, there is no genetic data available, but other information may still prove useful. Her cranial morphology shows some traits which are considered Mongoloid-type (see section 5.2.4.) and the facial depiction (Fig 1) also gives an impression of Inuit or Asian influence. These are common traits in the Scandinavian and northwest Russian areas where she is likely to have been born and, although her maternal influence may be traced back to continental Europe, her father could have come from the region where she was born and thus have provided input for her somewhat ambiguous appearance.

The immigrant history of this individual introduces two main challenges: firstly, why was she born in the northern parts of Europe to a mother who may have had her origin, or at least her ancestry, in central or western parts of Europe, possibly on the Iberian Peninsula? Secondly, why did she migrate at least 800 km and end up in Trondheim? If the girl was born in Russia, her maternal ancestors might have followed a route by sea or by both sea and land, via mainland Europe. However, given the possibility that the girl was born in northern Swedish-Finish territories, and especially if her birthplace was located somewhere in present day northwestern Russia, we cannot rule out the possibility that an ancestor had travelled eastward towards Constantinople and the Black Sea and from there northwards on the Russian rivers. International trade routes were well established at the time and this strengthened contact between Norway and the British Isles, as well as mainland Europe, especially some of the western Germanic territories along the Rhine. In the 13th century, such contacts expanded towards areas east of the Elbe and along the southern coast of the Baltic Sea. Trading contacts involved both foreign merchants visiting major Norwegian towns like Bergen, Trondheim and Oslo, as well as Norwegian traders visiting harbours in the British Isles, especially eastern English harbour towns, and harbours along the Baltic coast [81]. Exactly why this young girl travelled to Trondheim is impossible to establish, but the most likely route of travel would have been by ship southwards along the Norwegian coast. The trade with dried fish along this route down to Bergen, and further into continental Europe, was among the biggest Norwegian exports and Trondheim was an important harbour along this route. Travelling over land for such a long distance would have been difficult and too strenuous in these parts of the world and seems less likely. A young girl, probably not yet reached her teens, wouldn’t have had many options when arriving as a newcomer in a medieval town. If she did not have family to care for her, the most likely way to make a living for a young girl would have been taking work in one of the towns wealthier households. She would, however, not have been unique with respect to having travelled at such a young age. Hamre and Daux [5] has shown that approximately a third of the medieval individuals in that study had moved to town by the time they had reached early teens.

The complexity of this immigrant’s story comes to the fore when considering the cultural aspects of this young woman’s migration to Trondheim. What kind of cultural and social challenges would a young immigrant girl, or any immigrant coming to medieval Norway, meet? If she was born and had spent her childhood anywhere in the northern parts of Norway, Sweden or Finland, she would most likely have been familiar with the common languages and dialects within this area. If, however, she was born and raised further east in the Russian territories, she would likely have met a language barrier when migrating to Trondheim. She would also have crossed a religious barrier between the Orthodox and Catholic churches, though it is not known to what extent this represented any profound obstacle for integrating into a medieval Norwegian urban environment.

### 4.3. The woman of central European descent: SK 196 (Fig 2)

**Fig 2:**
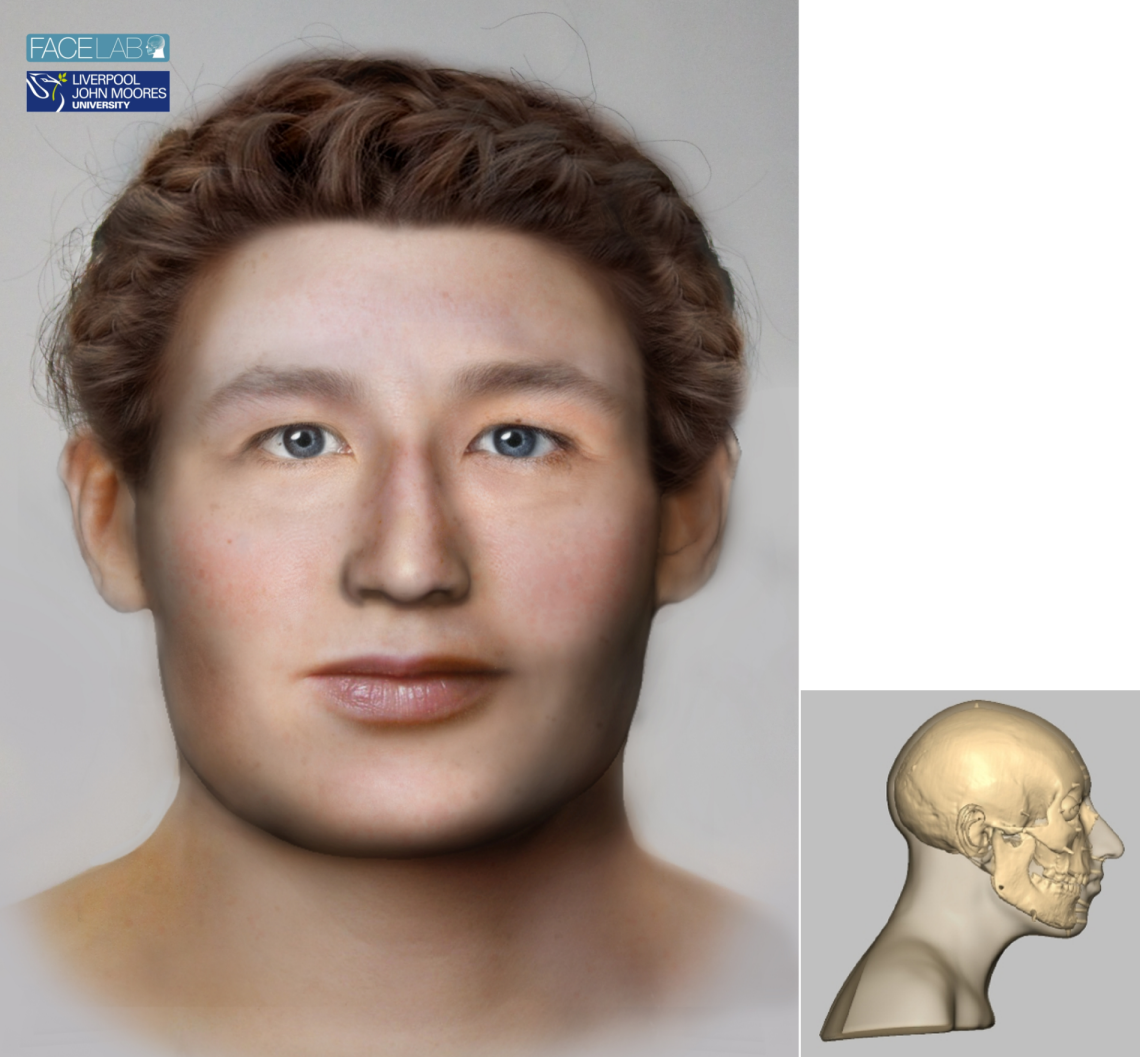
The left picture shows the facial depiction of SK196 while the right picture shows the profile view of the textured skull of the same woman.

#### 4.3.1. Sex, age at death, and stature 538

This person has also been determined to be female through morphological examination [7, 16], a conclusion recently confirmed by genetic sexing (Genderplex analysis). It has been estimated that this woman was slightly older than SK 152 at the time of death but she would not have lived long past her mid-twenties [7, 16]. Her living stature has been estimated on the basis of the 416mm maximum length of the left femur which indicates she was between 154.9 cm and 163.9 cm tall during her adult life [60].

#### 4.3.2. Geographic origins and movement

The δ^18^O values of the carbonates of the enamel from the first and third molars of this woman are 23.05‰ and 24.07‰ respectively. The isotopic composition of the M3 corresponds to an environmental water value at ~-10.4‰ (±1‰) which is consistent with late childhood/early teens spent in the area surrounding Trondheim [5]. The δ^18^O_w_ corresponding to the M1 tooth, corrected for a possible weaning effect and taking into account a maximum ±1‰ model error, is between about -14.6‰ and -11.4‰ [5], which gives several options for where this person was born. If she was born in Norway, the oxygen value is compatible with a geographic area from the coast of Nordland, 400–500 kilometres north of Trondheim, to an area about 200 kilometres east of Trondheim, towards the Swedish county of Jemtland (Norwegian territory at the time). Another option is that she was born in Sweden, on the coast of the Gulf of Bothnia. No matter where she was born, she moved during her childhood to an area with δ^18^O values compatible with an area close to Trondheim, although probably not to Trondheim itself. Areas in Russia, to the east of the Gulf of Bothnia, can also not be ruled out.

Another option, which may seem far-fetched at first glance, is that her origins lie in the central European Alps. For a δ^18^O_w_ range as depleted as that measured for her M1, the only option on the European mainland would be in the Alps at relatively high altitudes, probably more than 750 metres above sea level (e.g. [82, 83]). This possibility will be discussed in more detail below.

#### 4.3.3. Ancestry

The mtDNA CR haplotype of this woman was determined as 16177G 16189C 16223T 16292T 16362C 16519C 73G 150T 189G 194T 195C 204C 207A 263G 315.1C 523DEL 524DEL aligned against the rCRS [66, 68, 69]. This haplotype was not found in the largest forensic mtDNA database EMPOP (www.empop.online, R11; 70) nor in other online resources, suggesting that this haplotype is rare. This CR haplotype belongs to hg W5a2b. This hg is nested in super-hg W5a which is predominantly found in West Eurasian (Caucasian) individuals in Europe and North America (USA, descendants from European immigrants). EMPOP lists W5a matches in central Europe (Germany) and eastern Europe (Poland), while W5a2 is, so far, only found in Germany (http://www.eupedia.com/europe/Haplogroup_W_mtDNA.shtml).

#### 4.3.4. Cranial morphology and physical appearance

The skull was assessed morphologically and anthropometrically. The skull suggested a large, robust female with mild brow ridges, large cheek bones, a square jaw line, a wide palate, angular orbits, large mastoid processes, long facial distances and an upright profile. The skull was typically Caucasoid-type ancestry in skeletal characteristics, but was not typically female. Indeed, this skull showed many male characteristics that suggest the woman was a strong and robust individual.

The orbit morphology suggested horizontal eye fissures, deep-set eyes and central eyelid folds [40, 46, 50]. The shape of the brow and nasal root [52] suggested that SK196 had arched eyebrows. The nasal aperture shape [41, 71], nasal measurements [37], canine fossa [41] and nasal spine [41, 72] suggested that the nose of SK196 was long, with a straight dorsal ridge, horizontal columella, pointed nasal tip and no visible nasolabial creases. The teeth, occlusion and alveolar processes [41, 53] suggested that the lips of SK196 were moderately thick with up-turned corners, a narrow philtrum and lips of equal prominence. The skull of SK196 also suggested a rounded chin and square jaw line, and the mastoid processes [41], suggested that SK196 had large, lobed ears. The neck is determined by the position of the mastoid processes where the sternocleidomastoid muscles attach and SK196 showed a thick, muscular neck. For SK196, minimum European female tissue depth measurements were used to reflect a 13^th^ century diet and lifestyle.

Finally, the finished head is textured to add skin colour, eye colour, hair colour and style. Here the advice of experts at Bryggens museum in Bergen was considered using typical hairstyles. The DNA did not provide reliable information regarding the appearance of this individual and the choice of a pale, outdoors complexion, mid-brown hair and blue eye colour was based on what seemed most likely considering where she was found and the estimated birthplace. This part of the process was carried out in Face Lab using Adobe Photoshop.

The final head shows a robust and masculine face with wide features and a muscular neck.

#### 4.3.5. Pathology and trauma

**Fig 3:**
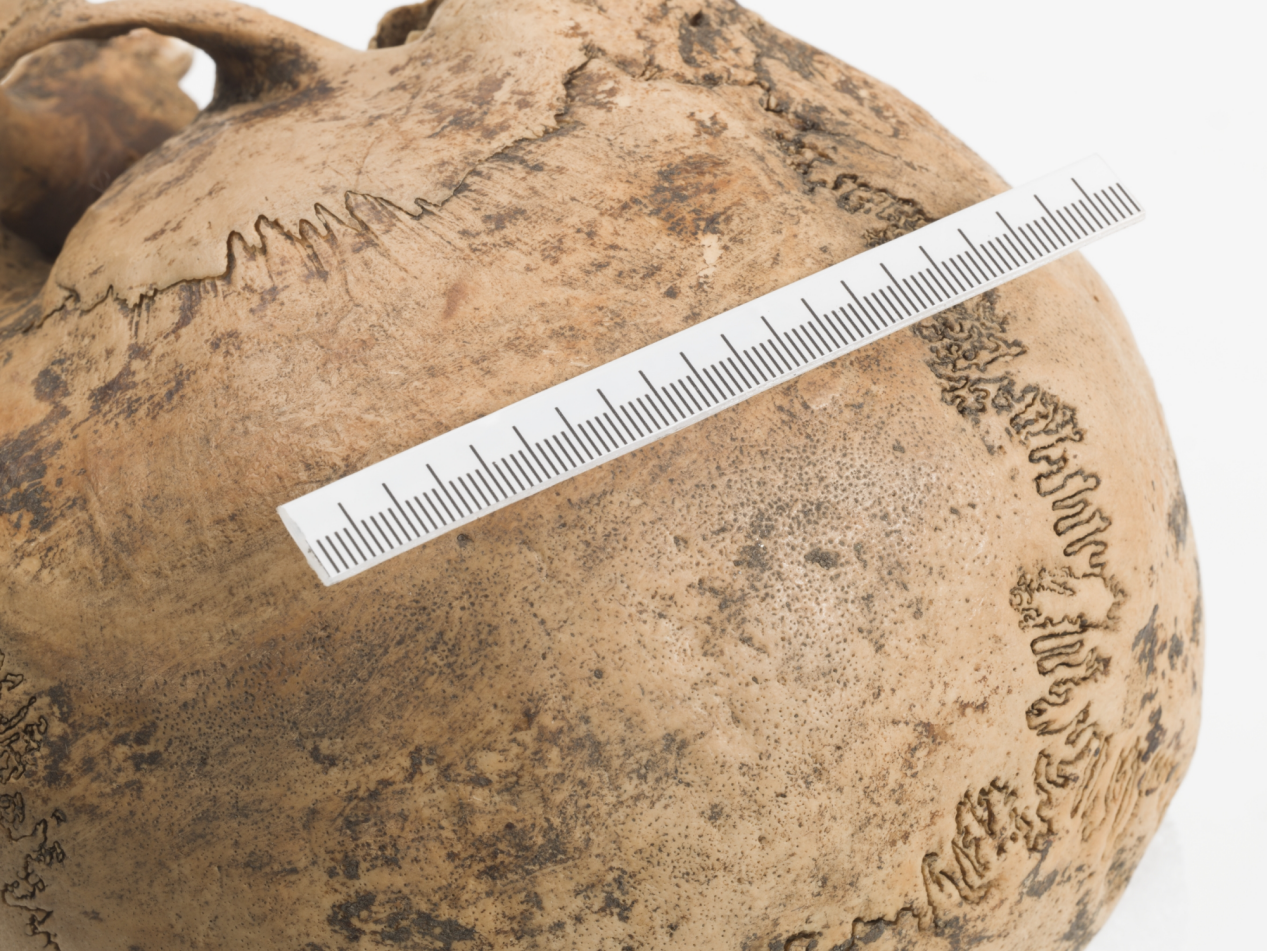
The right parietal of SK196 displaying signs of pitting indicative of porotic hyperostosis. Photo: Åge Hojem, NTNU Vitenskapsmuseet.

**Fig 4:**
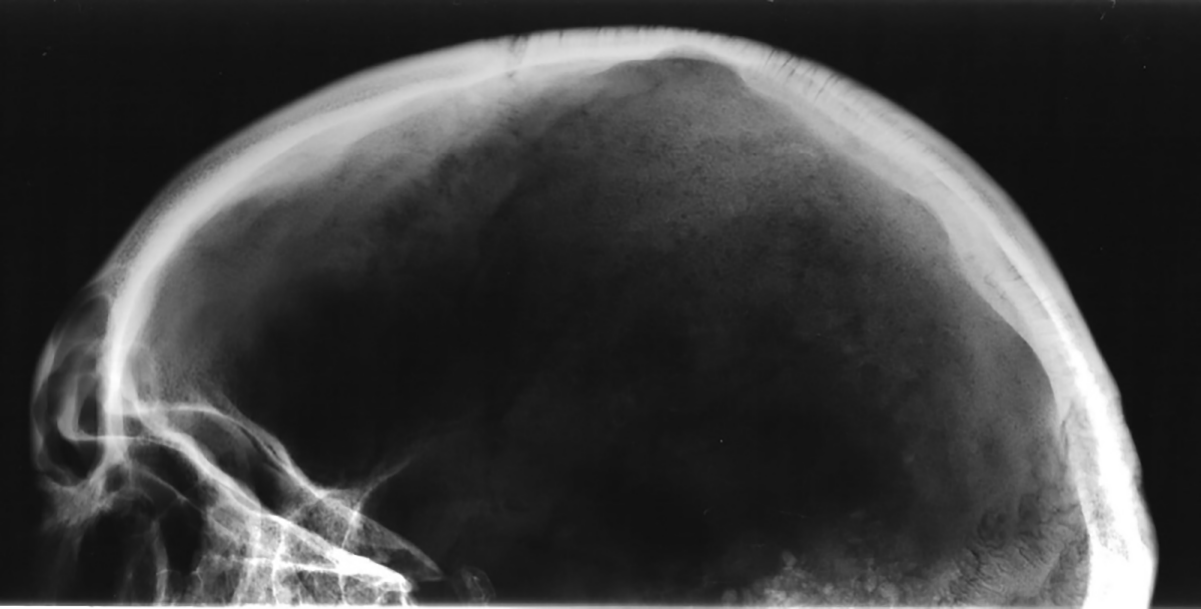
Left lateral view of the radiographed cranium of SK196 showing the characteristic “hair on end” pattern seen in porotic hyperostosis.

**Fig 5:**
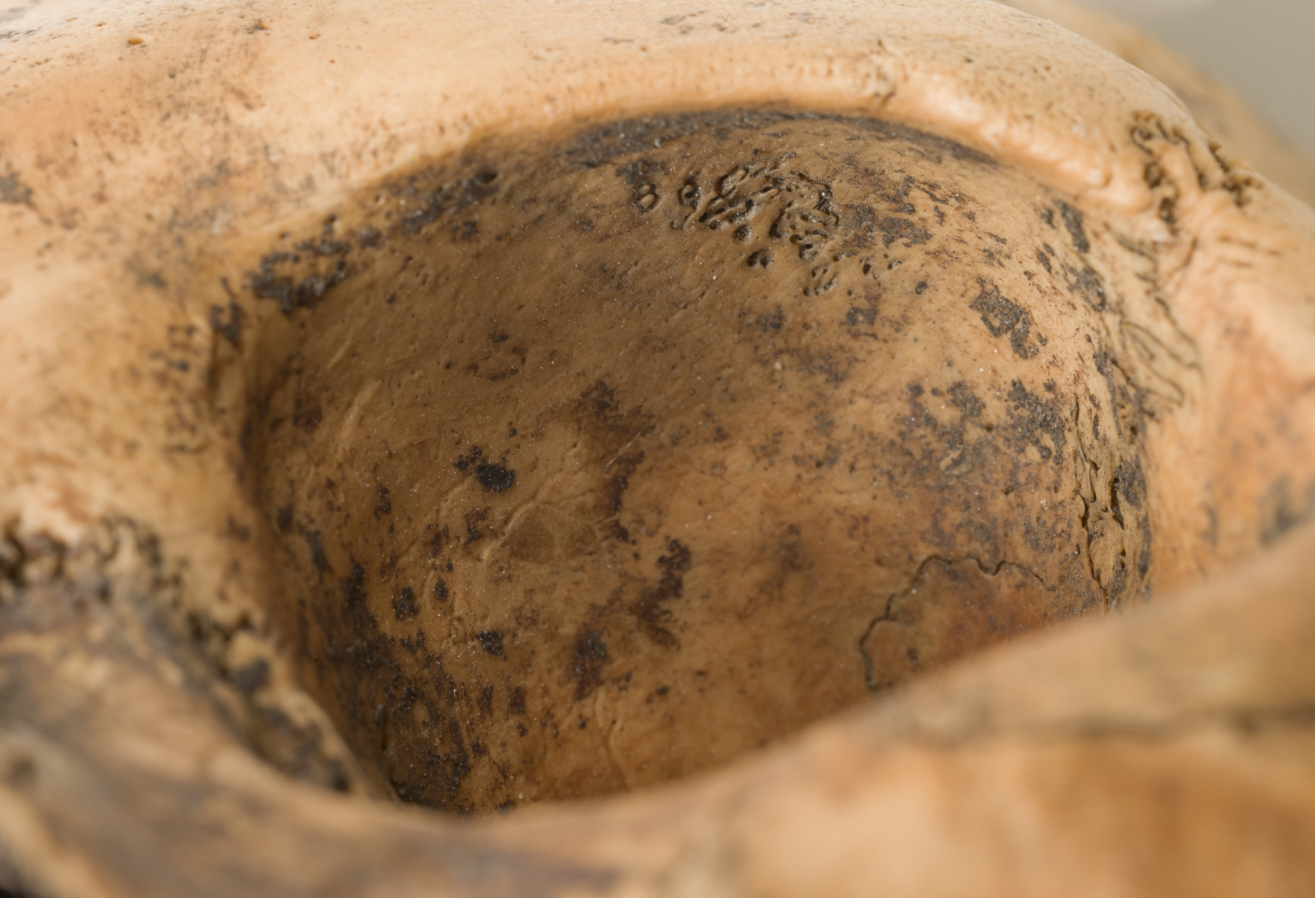
The roof of the left orbit of SK196 showing signs of cribra orbitalia. Photo: Åge Hojem, NTNU Vitenskapsmuseet.

This woman shows signs of porotic hyperostosis and cribra orbitalia. Porotic hyperostosis is identified macroscopically as areas of pitting and porosity on the outer surface of the cranial vault, while cribra orbitalia is identified as similar lesions on the roofs of the eye sockets. Such porosity is evident as a band stretching centrally across the right parietal bone from the lambdoid to the coronal suture (Fig 3). The pitting on the outer surface of the parietal bone is not markedly distinct and the lesions had healed for some time. The radiograph of the cranium (Fig 4) shows the characteristic “hair on end” pattern which testifies to the pitting being the consequence of an expansion of the diploic layer of the cranial vault, resulting in destruction of the outer table of the parietal bone. Partially healed cribra orbitalia lesions are also present on the roofs of both eye sockets (Fig 5).

The presence and aetiology of such lesions have been reported and discussed for over a century (e.g. [15, 84–88]) and there is still no definite agreement as to the underlying causes of these lesions. It is, however, generally agreed that anaemia is the main aetiological factor for porotic hyperostosis and an important cause of cribra orbitalia, although the orbital lesions seem to have a more varied aetiology [15]. It has also long been argued that iron deficiency is the type of anaemia which produces these lesions [89]. Walker et al. [15] have, however, presented a compelling argument against iron deficiency anaemia and put forward an equally convincing case for megaloblastic anaemia. The most common causes for this type of anaemia are vitamin B-12 and folate deficiency. Both deficiencies are related to diet: while B-12 is found in meat, fish, shellfish and dairy products, sources of folate include vegetables, particularly dark-green leafy vegetables, but also seafood, poultry and dairy products. Thus, a diet lacking such food sources could lead to megaloblastic anaemia, or a deficient mother could cause this to develop in her new born child through B-12- or folate-deficient breastmilk.

In the case of this woman, the lesions are well healed and she was not suffering from either deficiency at the time of death and had not done so for a number of years. Whether her anaemia stemmed from her mother, her own childhood condition, or both is difficult to say but it seems likely that her problems lasted well into her teens. Despite her likely deficiency, it does not seem she suffered periods of starvation or severe illness during growth as there are no signs of Harris lines on the radiograph of her tibia, nor of hypoplastic enamel on her teeth.

#### 4.3.6. Discussion

At first glance, the δ^18^O_W_ value between -14.6 and -11.4 for the first molar would suggest that this woman was born to the north or east of Trondheim, but combined with the mtDNA information, there is another possibility. The hg W5a2b, to which this woman belongs, is interesting in a Norwegian context, primarily for the reason that it is very rare. Hg W has a very low prevalence in modern day Norway (1.8%) and was the only hg present in modern Norway which was absent from the Viking age material in the largest study of Norwegian aDNA to date [6]. With this hg being so rare in Norway today, and never before found in pre-modern individuals in Norway, we are forced to consider the possibility that this woman was an immigrant herself or that her relatively near ancestors may have been. Her mtDNA suggests an origin in central Europe, possibly in Germany as that is the only place where the hg W5a2 is found (Eupedia: http://www.eupedia.com/europe/Haplogroup_W_mtDNA.shtml). Combining this information with the isotope values from her first and third molars, two scenarios seem possible.

First scenario is she was born in Norway with parents, or close ancestors, of central European descent. She would have spent her first years of life either on the coast of the county of Nordland or somewhere inland, between there and a region about 200 kilometres east of Trondheim. Her place of origin could have also been in Sweden on the coast of the Gulf of Bothnia.

The second scenario would be that she was born in central Europe and, if this was the case, the isotope value from her first molar makes it possible to pinpoint her birthplace with reasonable accuracy. The only places in central Europe where one may find δ^18^O_W_ values as low as -11.4 are in the Alps. Her third molar value is 2.12 points different from her birthplace value which means she lived in a different place in her late childhood/early teen years than where she spent her first few years of life. The δ^18^O_W_ value calculated from her third molar of -10.36‰ is, however, also a little low and does not leave too many options with regard to places she may have lived during her childhood years. It would have been enough to move to an area of lower altitude but still in mountainous regions as a δ^18^O_W_ value as depleted as -10.36 is not compatible with lowland areas in central Europe. Following this scenario, she would have moved to Trondheim as an adult. It is, of course, impossible to say with any certainty why she moved northwards to Trondheim. Did she move to Trondheim and settle down there or did she come to Trondheim with the intention of going back but happened to die there? Turning to historical sources may, however, shed light on some likely possibilities. There are two factors that might be considered more interesting than others. One is that she had followed the trading routes across Europe. The other major factor to be considered is that Trondheim was the centre of the Catholic church in Norway and a favourite destination for pilgrimage. The shrine of St. Olav was located in Trondheim and received many visitors, possibly in the thousands each year, mostly from the Nordic countries but also from areas further away, like central Europe. As a pilgrim, it is not unlikely that a young woman would visit Trondheim. If her illnesses still troubled her at the time she moved to Trondheim, she might have ventured to the shrine of St. Olav, since its healing powers were renowned. Otherwise, a change in eating habits, as a result of moving to Trondheim, will likely have cured her of her anaemia, as seafood would have formed a significant part of her Norwegian diet.

Culturally she would likely have been able to fit in considering the close connections through trade and religion. Still, she would have had to struggle with similar issues to those one would face today if moving to a different European country: differing day-to-day customs, different food, different climate, finding friends and work as a foreigner, and there would have been a language barrier she would have had to overcome. Bilingualism is likely to have been part of her skill set.

### 4.4. The man who needed surgery: SK 259 (Fig 6)

**Fig 6:**
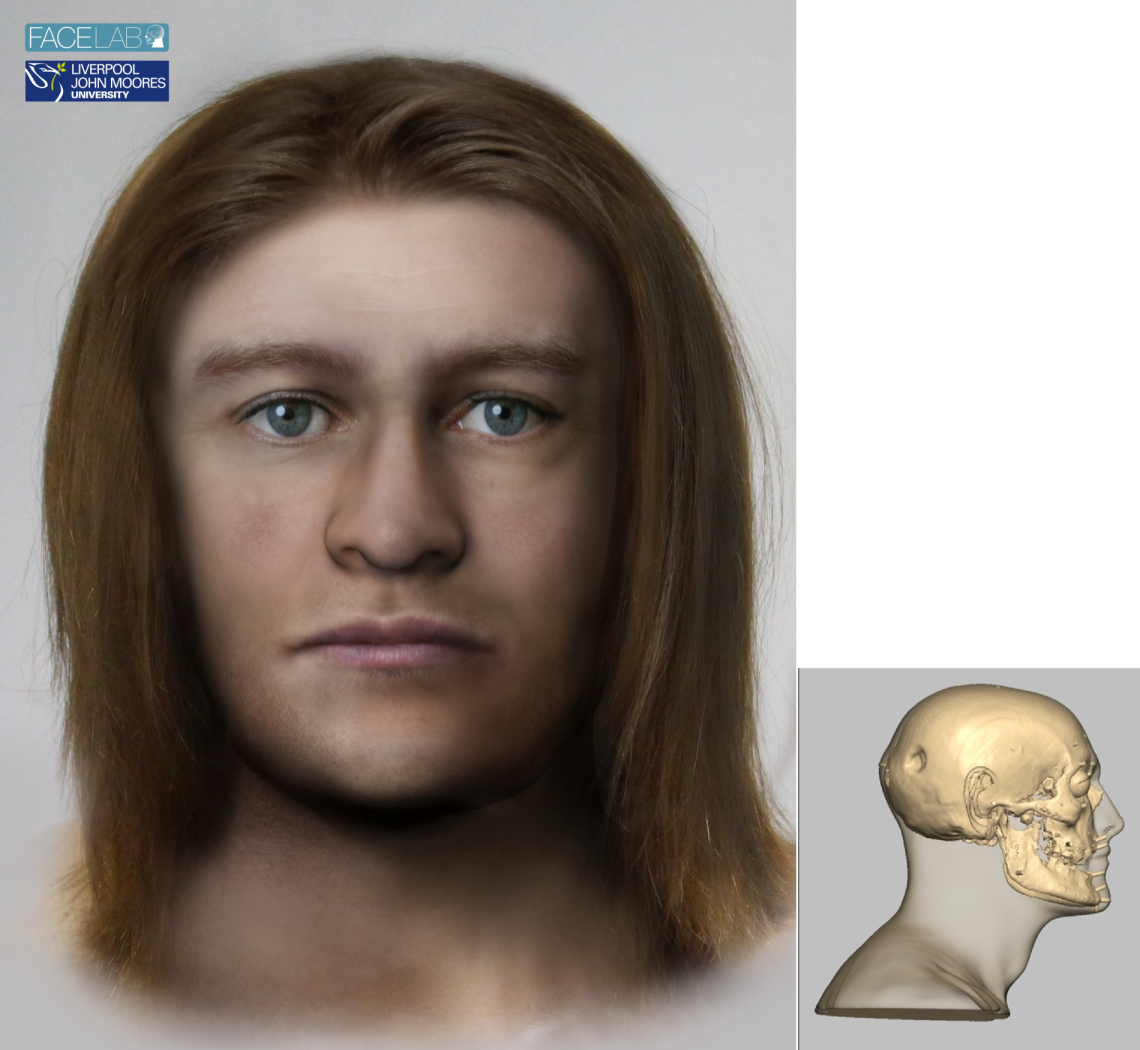
The left picture shows the facial depiction of SK259 while the right picture shows the profile view of the textured skull of the same man.

#### 4.4.1. Sex, age at death, and stature

The sex of this person was determined by morphological examination to be male [7, 16] and this has recently been confirmed by genetic sexing (Genderplex analysis). This person will have lived longer than the two women discussed in this paper, but not by much as he would have died in his late twenties or early thirties [7, 16]. His living stature has been estimated on the basis of the 456mm maximum length of the left femur which means he stood between 165.4 cm and 174.4 cm tall during his adult life [60].

#### 4.4.2. Geographic origins and movement

The δ^18^O values of the carbonates of the enamel from the first and third molars of this man are 23.88‰ and 23.97‰ respectively. The δ^18^O_w_ values consistent with the isotopic composition of the M1 are in the range of -10‰ to -13.2‰ which suggests that he was not born in Trondheim, but could have lived not very far away. In geographical terms, this means that this person was most likely born in one of the Norwegian counties of North or South Trøndelag, or possibly in southern Nordland. The Swedish county of Jämtland is equally possible. Moving slightly further away, the coastal areas of the Gulf of Bothnia are also a possibility. There is an overlap between the δ^18^O_w_ ranges for M1 and M3 (-10.52 ±1‰), so it is likely that he stayed in the same area in which he was born at least until he reached early adulthood.

#### 4.4.3. Ancestry

The mtDNA CR haplotype of this man was determined as 195C 263G 315.1C 523DEL 524DEL aligned against the rCRS [66, 68, 69]. This haplotype was found 15 times in the largest forensic mtDNA database EMPOP (www.empop.online, R11; [70]) with matches in West Eurasians (Germany, Baden-Württemberg (2x), Sweden, Blekinge and Värmland, and 11 times in the USA (descendants of immigrants). This CR haplotype is also frequently found in other online sources. Its haplotype is nested in haplogroup H, the most common haplogroup for West Eurasians.

The paternally inherited Y-Chromosomal haplotype is: DYS456 = 16; DYS389I = 13; DYS390 = 24; DYS389II = 29; DYS458 = 22; DYS19 = 14; DYS385 = 11,14; DYS393 = 13; DYS391 = 11; DYS439 = 12; DYS635 = 23; DYS392 = 13; YGATAH4 = 12; DYS437 = 15; DYS438 = 12; DYS448 = 19. It is found in the largest Y-Chromosomal forensic database (YHRD, www.yhrd.org) once in a western European individual living in the Bahamas; very likely a descendant of a European immigrant. The reduced haplotype (minHT) leads to 12 matches in Europe confirming the European descent of that haplotype.

#### 4.4.4. Cranial morphology and physical appearance

The skull was assessed morphologically and anthropometrically. The SK259 skull suggested a large, robust male with moderate brow ridges, a square jaw line, a wide palate, round orbits, strong cheek bones and an upright profile. The SK259 skull was typically male and Caucasoid-type in skeletal characteristics.

The orbit morphology suggested slightly upward sloping (laterally) eye fissures, prominent eyeballs and central eyelid folds [40, 46, 50] and the shape of the brow and nasal root [52] suggested that SK259 had low, straight eyebrows. The nasal aperture shape [41, 71], nasal measurements [37], canine fossa [41] and nasal spine [41, 72] suggested that the nose of SK259 had an up-turned columella, straight dorsal ridge, flat nasal tip and no visible nasolabial creases. The teeth, occlusion and alveolar processes [41, 53] suggested that the lips were moderately thick with up-turned corners, a wide philtrum and lips of equal prominence. The skull of SK259 also suggested a prominent chin and square jaw line. The mastoid processes [41], suggested that this man had large, lobed ears. The neck, determined by the position of the mastoid processes where the sternocleidomastoid muscles attach, showed a thick, muscular neck. Minimum European male tissue depth measurements were used to reflect a 13^th^ century diet and lifestyle.

Finally, the finished head is textured to add skin colour, eye colour, hair colour and style. Here the advice of the experts at Bryggens museum in Bergen was considered using typical hairstyles. The DNA did not provide reliable information regarding the appearance of this individual and the choice of light skin, dark blonde hair and blue eye colour was based on what seemed most likely considering where he was found and the estimated birthplace. This part of the process was carried out in Face Lab using Adobe Photoshop and a 3D stereolithographic copy of the head was also produced for the museum exhibition. The final head showed a robust male face with large features.

#### 4.4.5. Pathology and trauma

**Fig 7:**
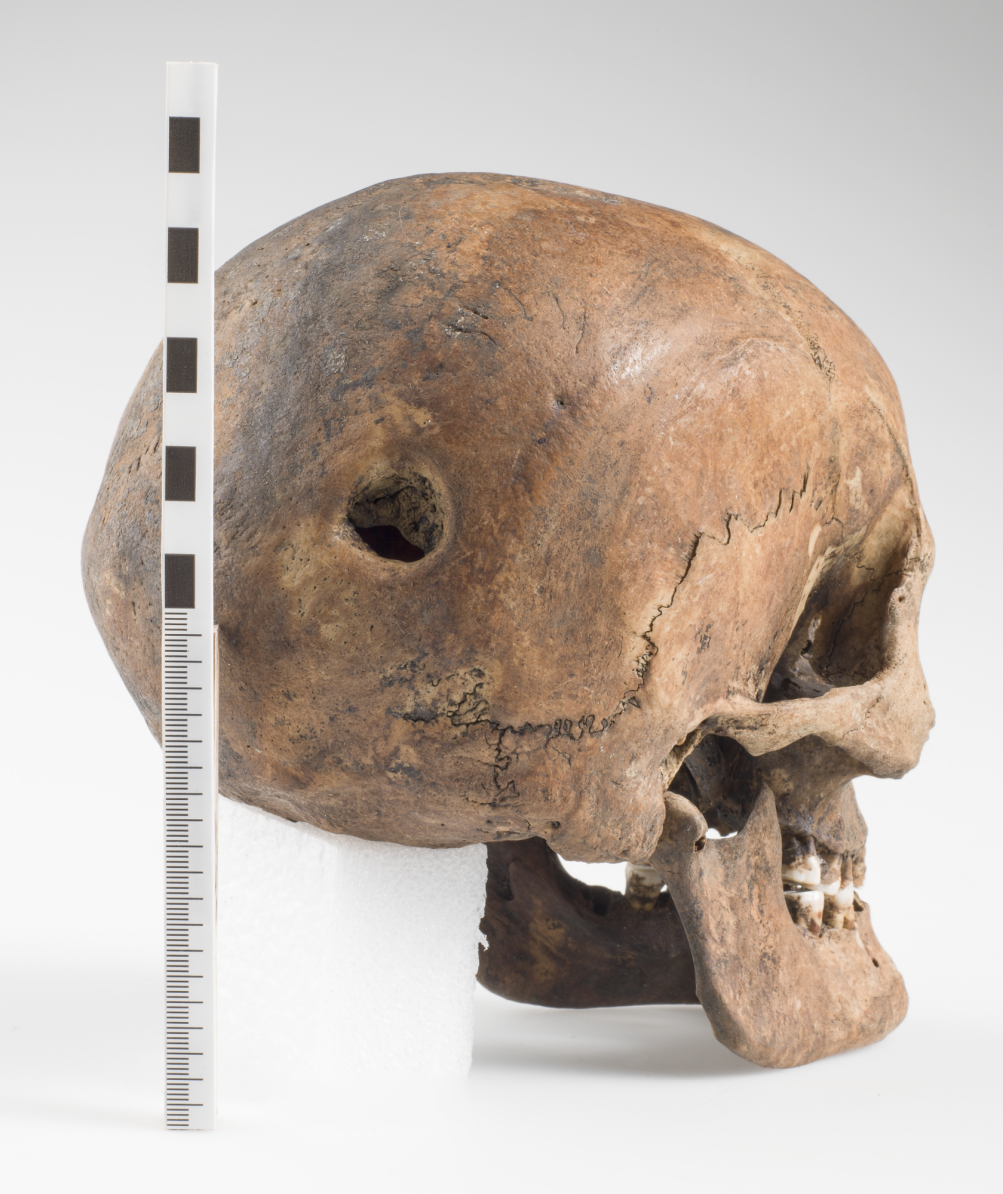
The skull of SK259 showing the trepanation hole in the posterior part of the right parietal bone. Photo: Åge Hojem, NTNU Vitenskapsmuseet.

**Fig 8:**
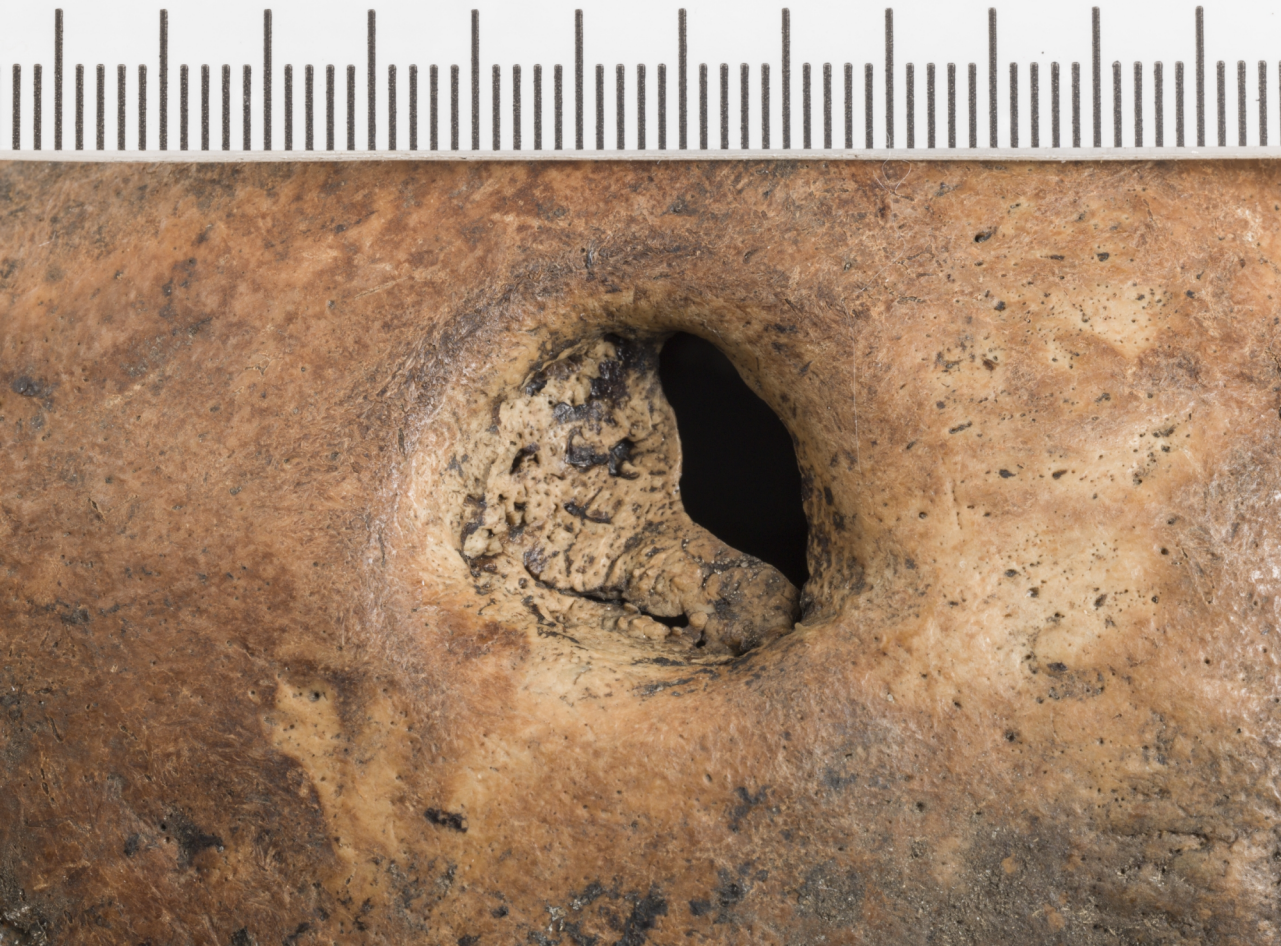
Close up of the trepanation in the cranium of SK259 showing the bone was healing well. Photo: Åge Hojem, NTNU Vitenskapsmuseet.

This person had undergone a surgical procedure as is evident by the trepanation in the posterior part of the right parietal bone (Fig 7). The trepanation is circular with a diameter of ca. 22 mm. The procedure was carried out a relatively long time before his death judging from the rounded edges of the trepanation hole. The inner layer of compact cranial bone has grown to cover two thirds of the opening (Fig 8) which suggests that he survived for months rather than weeks. This testifies to the presence of a highly skilled surgeon in Trondheim during the 13th century. Specialised skills are required to perform successful surgery which involves opening the scalp and then drilling a hole in the cranium with such precision as not to damage the meninges, which would have been fatal, and at the same time making sure the patient did not contract an infection and that the wound did not become dangerously septic. Who could have had the necessary skills to perform such a procedure?

This was not an everyday event in medieval Trondheim or in Norway, or in Scandinavia for that matter. This is the only known case of successful trepanation in medieval Norway. Holck [90] presents two cases of possible, or partial, trepanation from medieval Hamar in Norway, both rather different from the case presented here. Both cases described by Holck [90] are circular incisions located near the bregma but neither has fully penetrated the cranial bone. In one case, the cut goes through the diploe but not the inner layer of compact bone, while the other case of attempted trepanation has only left a shallow circular groove in the outer layer of compact cranial bone. Thus, the case of SK259 from Trondheim, is the only recorded example of a completed trepanation from medieval Norway. The only other case of a likely trepanation is found on a Neolithic skull from Nyelv in Finnmark, found in 1939 [91–93]. Jennbert [94] lists 29 known trepanations, both complete and incomplete, from Sweden dating from the Neolithic to medieval times. Eleven of the trepanations date from the medieval period but only five of these are complete trepanations. All five show signs of healing, testifying to the patients having survived the surgery. In Denmark, there are 18 reported cases of trepanation dating from the Neolithic to the Iron Age but several of these should possibly be diagnosed differently [95]. None of the Danish examples date from the medieval period and thus, the total number of completed trepanations from medieval Scandinavia is only six. Considering there are thousands of excavated medieval skeletons from Scandinavia, and only six known, successfully performed, trepanations, suggests that being trepanned in medieval Scandinavia was a very rare event.

**Fig 9:**
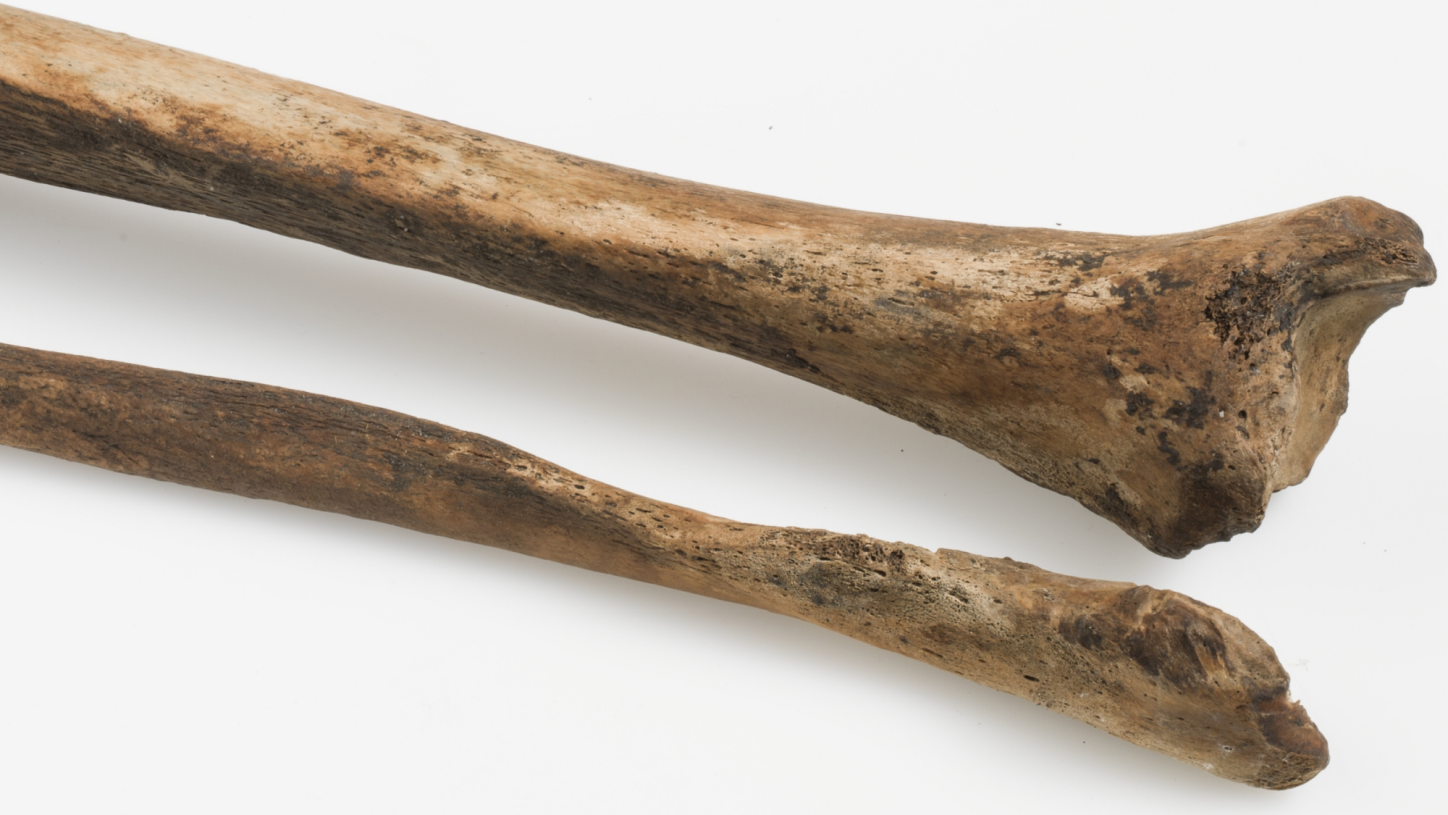
The right tibia and fibula of SK259 showing periostitis on the central and distal parts of the diaphysis of both bones. Similar lesions are also found on the left tibia and fibula. Photo: Åge Hojem, NTNU Vitenskapsmuseet.

Trepanations were mainly used as treatment for head injuries by draining blood from intracranial haematomas, or removing fractured cranial bone fragments or necrotic bone [96, 97]. This use was also described by the most respected physician in Western antiquity, Galen of Pergamum [98], and in the Hippocratic texts [96]. In this case, however, head trauma may not be the likeliest reason for the trepanation as there is no evidence of injury on this skull. A suspected intracranial haematoma due to a non-fracturing head trauma could still have been the reason for the trepan, but an underlying disease is more likely. The reason for this assertion is the pathological changes present on both tibiae and fibulae (Fig 9). There are signs of periostitis on large areas of the central and distal parts of both tibiae and fibulae. Bilateral, localised, periosteal inflammation like this could be the result of an indeterminable systemic infection. These lesions could also be seen in tuberculosis [11] but are rare in adults and, without any other signs of pathological changes on the skeleton, this seems unlikely. A condition which causes localised periostitis in the lower legs is venous insufficiency [99, 100] which is a condition where the flow of blood through the veins is inadequate, causing blood to pool in the legs. The aetiology for venous insufficiency can be blood clots or varicose veins, and occupations involving long periods of time standing may result in chronic venous distention and secondary valvular incompetence [100]. Whatever the underlying cause, if this was the condition he suffered from, this person would have suffered discomfort in the legs, possibly to a debilitating extent. Symptoms would have included several of the following: swelling of the legs, pain, leg cramps, feeling of heaviness in the legs, itching, weak legs, thickening and pigmentation of the skin. If this is left untreated and the symptoms are not reversed, this condition may lead to ulceration. Prolonged inflammation in the soft tissues may increase the blood flow to the underlying periosteum, which can then produce new subperiosteal bone [100]. In what way, if at all, this relates to the trepanation is very difficult to say. This underlying pathological condition does, however, suggest that the reason for the trepanation was a disease, rather than trauma or mental illness, which is also likely to have been treated with this kind of cranial surgery [96, 97].

#### 4.4.6. Discussion

The birthplace of this person puts him among the majority of the population in medieval Trondheim as he was born outside the town but probably not too far away [5]. His hg H is very common in Europe, including the suggested areas of birth for this individual, so there is no reason to suggest that his ancestors came from somewhere else. It is unsurprising to find that this individual came from the neighbouring rural districts. As in medieval Europe in general, towns and cities provided employment opportunities and attracted people from the countryside. Such attraction even caused workforce shortages in rural medieval Norway and the King issued laws against people taking up petty trade instead of tending to farming. Seeking work might be the most obvious possible reason when trying to explain why this individual ended his life in Trondheim. However, since he had been treated for his illnesses and undergone surgery, this might also have been the reason for his move. It was most likely among the clergy in Trondheim that the knowledge and skills to perform such treatment was to be found and the presence of such expertise may have been well-known among the local population. Alternatively, it is possible that the healing abilities attributed to the shrine of St. Olav were the factor that drew the patient to Trondheim.

## 5. Conclusion

The three life stories outlined above give an insight into the diversity of Norwegian medieval society. The three individuals were all of average social status and, although they were not chosen completely at random, they represent normal members of society and their stories will not have been unique. As shown by Hamre and Daux [5], the medieval population in Trondheim was a mobile one and consisted of people of varied geographic origins. The three individuals presented in this article represent this population and give an insight into individual lives in one of the major towns in medieval Norway. They are representatives of a rather heterogeneous population. This is not surprising as it is well known that these towns were urban environments. As the seat of an archdiocese, Trondheim was one of the metropoles of the papal church and was a nodal point on long distant routes. People travelled along these routes for a variety of reasons, some for shorter distances, others through larger stretches of Europe. The town would have attracted people from surrounding areas seeking work, as well as tradespeople and skilled craftsmen. Trondheim was also a popular destination for pilgrims coming to visit the shrine of St. Olav. From early on, the medieval towns in Norway became places where people with different cultural backgrounds, different customs and different languages lived together. The heterogeneity seen in modern Norwegian cities can be traced back to the early medieval towns; urban environments which have developed into present-day societies.

## References

[1] Christophersen A, Nordeide SW. Kaupangen ved Nidelva. Trondheim: Riksantikvaren; 1994 324 p.

[2] Castro MEA, Waters-Rist AL, Zborover D. An osteobiography of a Oaxacan late adolescent female. Journal of Archaeological Science: Reports. 2016.

[3] Bagge S. Cross and Scepter: The Rise of the Scandinavian Kingdoms from the Vikings to the Reformation. Princeton New Jersey: Princeton University Press; 2014.

[4] Helle K. The Norwegian kingdom: succession disputes and consolidation In: Helle K, editor. The Cambridge History of Scandinavia Prehistory to 1520. 1. Cambridge: Cambridge University Press; 2003 p. 369–91.

[5] Hamre SS, Daux V. Stable oxygen isotope evidence for mobility in medieval and post-medieval Trondheim, Norway. Journal of Archaeological Science: Reports. 2016;8:416–25.

[6] Krzewinska M, Bjørnstad G, Skoglund P, Olason PI, Bill J, Götherström A, et al Mitochondrial DNA variation in the Viking age population of Norway. Philosophical Transactions of the Royal Society B. 2015;370(http://rstb.royalsocietypublishing.org/content/370/1660/20130384). 10.1098/rstb.2013.0384 PMC4275891 25487335

[7] Anderson T, Göthberg H. Fortiden i Trondheims bygrunn: Folkebibliotekstomten. Olavskirkens kirkegård. Human-osteologisk analyse og faseinndeling. Trondheim: Riksantikvaren, utgravningskontoret for Trondheim; 1986. Report No.: 2.

[8] Christophersen A. Strete, havn og kirkegård In: Christophersen A, Nordeide SW, editors. Kaupangen ved Nidelva. Riksantikvarens Skrifter. Trondheim: Riksantikvaren; 1994 p. 324.

[9] Nordeide SW. St. Olavs by. In: Christophersen A, editor. Fra Nidarnes til Trondheim Vandringer i et Bylandskap. Trondheim: NINA-NIKU; 1997 p. 48–59.

[10] Alfonso MP, Thompson JL, Standen VG. Reevaluating Harris lines—A comparison between Harris lines and enamel hypoplasia. Collegium Antropologicum. 2005;29(2):393–408. 16417135

[11] Aufderheide AC, Rodriguez-Martin C. The Cambridge encyclopedia of human paleopathology. Cambridge: Cambridge University Press; 1998.

[12] Callear TE. Lines and bands of increased density. Their implication to growth and development. Medical Radiography and Photography. 1968;44:58–89. 5747254

[13] Mays S. The relationship between Harris lines and other aspects of skeletal development in adults and juveniles. Journal of Archaeological Science. 1995;22:511–20.

[14] Stuart-MacAdam P. A radiographic study of porotic hyperostosis. American Journal of Physical Anthropology. 1987;74:511–20. doi: 10.1002/ajpa.1330740409 3327383 10.1002/ajpa.1330740409

[15] Walker PL, Bathurst RR, Richman R, Gjerdrum T, Andrushko VA. The causes of porotic hyperostostosis and cribra orbitalia: A reappraisal of the iron-deficiency-anemia hypothesis. American Journal of Physical Anthropology. 2009;139:109–25. doi: 10.1002/ajpa.21031 19280675 10.1002/ajpa.21031

[16] Hamre S. Burial Practices in Early Christian Norway An Osteoarchaeological Study Into Differences and Similarities Between Four Burial Assemblages. Bergen: University of Bergen; 2011.

[17] Daux V, Lécuyer C, Héran MA, Amiot R, Simon L, Fourel F, et al Oxygen isotope fractionation between human phosphate and water revisited. Journal of Human Evolution. 2008;55(6):1138–47. doi: 10.1016/j.jhevol.2008.06.006 18721999 10.1016/j.jhevol.2008.06.006

[18] Chenery CA, Pashley V, Lamb AL, Sloane HJ, Evans JA. The oxygen isotope relationship between the phosphate and structural carbonate fractions of human bioapatite. Rapid Communications in Mass Spectrometry. 2012;26(3):309–19. doi: 10.1002/rcm.5331 22223318 10.1002/rcm.5331

[19] Evans JA, Chenery C, Fitzpatrick AP. Bronze age childhood migration of individuals near stonehenge, revealed by strontium and oxygen isotope tooth enamel analysis. Archaeometry. 2006;48(2):309–21.

[20] Wright LE, Schwarcz HP. Stable carbon and oxygen isotopes in human tooth enamel: identifying breastfeeding and weaning in prehistory. American Journal of Physical Anthropology. 1998;106:1–18. doi: 10.1002/(SICI)1096-8644(199805)106:1<1::AID-AJPA1>3.0.CO;2-W 9590521 10.1002/(SICI)1096-8644(199805)106:1<1::AID-AJPA1>3.0.CO;2-W

[21] Wright LE, Schwarcz HP. Correspondence Between Stable Carbon, Oxygen and Nitrogen Isotopes in Human Tooth Enamel and Dentine: Infant Diets at Kaminaljuyú. Journal of Archaeological Science. 1999;26(9):1159–70.

[22] Shahar S. Childhood in the middle ages. London: Routledge; 1990.

[23] Dansgaard W. Stable isotopes in precipitation. Tellus. 1964;XVI(4):436–68.

[24] Esper J, Frank DC, Timonen M, Zorita E, Wilson RJS, Luterbacher J, et al Orbital forcing of tree-ring data. Nature Climate Change. 2012;2:862–6.

[25] Gunarson BE, Linderholm HW, Moberg A. Impreoving a tree-ring reconstruction from west-central Scandinavia: 900 years of warm-season temperatures. Climate Dynamics. 2011;36:97–108.

[26] Zhang P, Linderholm HW, Gunnarson BE, Björklund J, Chen D. 1200 years of warm-season temperature variability in central Scandinavia inferred from tree-ring density Climate of the Past 2016;12:1297–312.

[27] Amory S, Huel R, Bilic A, Loreille O, Parsons TJ. Automatable full demineralization DNA extraction procedure from degraded skeletal remains. Forensic Science International: Genetics. 2012;6:398–406. 21885362 10.1016/j.fsigen.2011.08.004

[28] Bauer CM, Niederstätter H, McGlynn G, Stadler H, Parson W. Comparison of morphological and molecular genetic sex-typing on mediaeval human skeletal remains. Forensic Science International: Genetics. 2013;7(6):581–6. 23941903 10.1016/j.fsigen.2013.05.005 PMC3820020

[29] Niederstätter H, Köchl S, Grubwieser P, Pavlic M, Steinlechner M, Parson W. A modular real-time PCR concept for determining the quantity and quality of human nuclear and mitochondrial DNA. Forensic Science International: Genetics. 2007;1(1):29–34. 19083725 10.1016/j.fsigen.2006.10.007

[30] Walker JA, Hedges DJ, Perodeau BP, Landry KE, Stoilova N, Laborde ME, et al Multiplex polymerase chain reaction for simultaneous quantitation of human nuclear, mitochondrial, and male Y-chromosome DNA: application in human identification. Analytical Biochemistry. 2005;337(1):89–97. doi: 10.1016/j.ab.2004.09.036 15649380 10.1016/j.ab.2004.09.036

[31] Esteve Codina A, Niederstätter H, Parson W. “GenderPlex” a PCR multiplex for reliable gender determination of degraded human DNA samples and complex gender constellations. International Journal of Legal Medicine. 2009;123(6):459–64. doi: 10.1007/s00414-008-0301-z 19089439 10.1007/s00414-008-0301-z

[32] Athey TW. Haplogroup Prediction from Y-STR Values Using an Allele-Frequency Approach. Journal of Genetic Genealogy. 2005;1(1):1–7.

[33] Athey TW. Haplogroup Prediction from Y-STR Values Using a Bayesian-Allele-Frequency Approach. Journal of Genetic Genealogy. 2006;2(2):34–9.

[34] Berger C, Parson W. Mini-midi-mito: Adapting the amplification and sequencing strategy of mtDNA to the degradation state of crime scene samples. Forensic Science International: Genetics. 2009;3(3):149–53. 19414161 10.1016/j.fsigen.2009.01.011

[35] Wilkinson CM. Facial Anthropology and Reconstruction In: Thompson TJK, Black SJ, editors. Forensic Human Identification. Boca Ratan: CRC Press; 2006 p. 231–56.

[36] Kau CN, Zhurov A, Richmond S, Cronin A, Savio C, Mallorie C. Facial templates: a new perspective in three dimensions. Orthodontics & Craniofacial Research. 2006;9:10–7. 16420270 10.1111/j.1601-6343.2006.00359.x

[37] Rynn C, Balueva T, Veselovskaya E. Relationships between the skull and face In: Wilkinson CM, R C., editors. Craniofacial Identification. Cambridge: Cambridge University Press; 2012 p. 193–202.

[38] Wilkinson CM. Forensic Facial Reconstruction. Cambridge: Cambridge University Press; 2004.

[39] Wilkinson CM. Facial reconstruction-anatomical art or artistic anatomy? Journal of Anatomy. 2010;216:235–50. doi: 10.1111/j.1469-7580.2009.01182.x 20447245 10.1111/j.1469-7580.2009.01182.x PMC2815945

[40] Angel J, editor Restoration of head and face for identification. Proceedings of Meetings of American Academy of Forensic Science; 1978; St Louis, USA.

[41] Gerasimov M. The reconstruction of the face from the basic structure of the skull. Moscow: Nauka; 1955.

[42] Krogman WM, Iscan MY. The Human Skeleton in Forensic Medicine. 2nd ed Springfield: Charles C Thomas; 1986.

[43] Stephan CN, Davidson PL. The placement of the human eyeball and canthi in craniofacial identification. Journal of Forensic Sciences. 2008;53:612–9. doi: 10.1111/j.1556-4029.2008.00718.x 18471206 10.1111/j.1556-4029.2008.00718.x

[44] Stephan CN, Murphy S. Mouth width prediction in craniofacial identification: cadaver tests of four recent methods, including two techniques for edentulous skulls. Journal of Forensic Odonto-Stomatology. 2008;27:2–7. 22689350

[45] Stewart T. The points of attachment of the palpebral ligaments: their use in facial reconstructions on the skull. Journal of Forensic Sciences. 1983;28:858–63. 6631367

[46] Whitnall SE. The Anatomy of the Human Orbit. Oxford Medical Publications London 1921.

[47] Prokopec M, Ubelaker DH. Reconstructing the shape of the nose according to the skull. Forensic Science Communications. 2002;4(1).

[48] Schultz AH. Relation of the external nose to the bony nose and nasal cartilages in whites and negroes. American Journal of Physical Anthropology. 2005;1:329–38.

[49] Stephan CN. Facial approximation: globe projection guideline falsified by exophthalmometry literature. Journal of Forensic Sciences. 2002;47:730–5. 12136981

[50] Wilkinson CM, Mautner SA. Measurement of eyeball protrusion and its application in facial reconstruction. Journal of Forensic Sciences. 2003;48:12–6. 12570193

[51] Balueva TS, Veselovskaya EV. New developments in facial reconstruction. Archaeology, Ethnology and Anthropology of Eurasia. 2004(1):143–52.

[52] Fedosyutkin BA, Nainys JV. The relationship of skull morphology to facial features In: İşcan MY, Helmer RP, editors. Forensic Analysis of the Skull. New York: Wiley-Liss; 1993 p. 199–213.

[53] Wilkinson CM, Motwani M, Chiang E. The relationship between the soft tissues and the skeletal detail of the mouth. Journal of Forensic Sciences. 2003;48:728–32. 12877287

[54] Wilkinson CM, Rynn C. Craniofacial Identification. Cambridge: Cambridge University Press; 2012.

[55] Lee WJ, Wilkinson CM, Hwang HS. An Accuracy Assessment of Forensic Computerized Facial Reconstruction Employing Cone-Beam Computed Tomography from Live Subjects. Journal of forensic sciences. 2012;57(2):318–27. doi: 10.1111/j.1556-4029.2011.01971.x 22073932 10.1111/j.1556-4029.2011.01971.x

[56] Wilkinson CM, Rynn C, Peters H, Taister M, Kau CH, Richmond S. A blind accuracy assessment of computer-modeled forensic facial reconstruction using computed tomography data from live subjects. Forensic Science, Medicine, and Pathology. 2006;2:179–87. doi: 10.1007/s12024-006-0007-9 25868696 10.1007/s12024-006-0007-9

[57] Landro T. Dei Eldste Norske Kristenrettane Innhald og Opphav. Bergen: University of Bergen; 2005.

[58] Landro T. Kristenrett og Kyrkjerett. Borgartingsretten i et Komparativt Perspektiv. Bergen: University of Bergen; 2010.

[59] Walter BS, DeWitte SN. Urban and rural mortality and survival in Medieval England. Annals of Human Biology. 2017. 10.1080/03014460.2016.1275792 28006969

[60] Sjøvold T. Estimation of stature from long bones utilizing the line of organic correlation. Human Evolution. 1990;5(5):431–47.

[61] Kortelainen N. Isotopic fingerprints in surficial waters: Stable isotope methods applied in hydrogeological studie: University of Helsinki; 2007.

[62] Kurita N, Yoshida N, Inoue G, Chayanova EA. Modern isotope climatology of Russia: A first assessment. Journal of Geophysical Research. 2004;109:D03102.

[63] Frits P, Drimmie RJ, Frape SK, O'Shea K. The isotopic composition of precipitation and groundwater in Canada International symposium on the use of isotope techniques in water resources development; IAEA, Vienna (Austria) Vienna: IAEA; 1987 p. 539–50.

[64] Fitzhugh WF, Ward E, editors. Vikings: The North Atlantic Saga Smithsonian Books; 2000.

[65] Seaver KA. The Frozen Echo: Greenland and the Exploration of North America, ca. A.D. 1000–1500 Stanford University Press; 1997.

[66] Andrews RM, Kubacka I, Chinnery PF, Lightowlers RN, Turnbull DM, Howell N. Reanalysis and revision of the Cambridge reference sequence for human mitochondrial DNA. Nature Genetics. 1999; 23(2). 10.1038/13779 10508508

[67] Anderson S, Bankier AT, Barrell BG, de Bruijn MH, Coulson AR, Drouin J, et al Sequence and organization of the human mitochondrial genome. Nature. 1981;290(5806):457–65. 7219534 10.1038/290457a0

[68] Bandelt HJ, Parson W. Consistent treatment of length variants in the human mtDNA control region: a reappraisal. International Journal of Legal Medicine. 2008;122(1):11–21. doi: 10.1007/s00414-006-0151-5 17347766 10.1007/s00414-006-0151-5

[69] Parson W, Gusmao L, Hares DR, Irwin JA, Mayr WR, Morling N, et al DNA Commission of the International Society for Forensic Genetics: revised and extended guidelines for mitochondrial DNA typing. Forensic Science International: Genetics. 2014;13:134–42. 25117402 10.1016/j.fsigen.2014.07.010

[70] Parson W, Dür A. EMPOP-a forensic mtDNA database. Forensic Science International: Genetics. 2007;1(2):88–92. 19083735 10.1016/j.fsigen.2007.01.018

[71] Rynn C. Craniofacial approximation and reconstruction: Tissue depth patterning and the prediction of the nose: University of Dundee; 2006.

[72] Rynn C, Wilkinson CM, Peters HL. Prediction of nasal morphology from the skull. Forensic Science, Medicine, and Pathology. 2010;6:20–34. doi: 10.1007/s12024-009-9124-6 19924578 10.1007/s12024-009-9124-6

[73] Denis KL, Speidel TM. Comparison of three methods of profile change prediction in the adult orthodontic patient. American Journal of Orthodontics and Dentofacial Orthopedics. 1987;92:396–402. 3479007 10.1016/0889-5406(87)90260-5

[74] Holdaway R. A soft-tissue cephalometric analysis and its use in orthodontic treatment planning. Part I. American Journal of Orthodontics. 1983;84: 1–28. 6575614 10.1016/0002-9416(83)90144-6

[75] Koch R, Gonzales A, Witt E. Profile and soft tissue changes during and after orthodontic treatment. The European Journal of Orthodontics. 1979;1:193–9. 296943 10.1093/ejo/1.3.193

[76] Roos N. Soft-tissue profile changes in Class II treatment. American Journal of Orthodontics. 1977;72:165–75. 268147 10.1016/0002-9416(77)90057-4

[77] Rudee DA. Proportional profile changes concurrent with orthodontic therapy. American Journal of Orthodontics. 1964;50:421–34.

[78] Talass MF, Tollaae L, Baker RC. Soft-tissue profile changes resulting from retraction of maxillary incisors. American Journal of Orthodontics and Dentofacial Orthopedics. 1987;91:385–94. 3472457 10.1016/0889-5406(87)90391-x

[79] Waldman BH. Change in lip contour with maxillary incisor retraction. The Angle Orthodontist. 1982;52:129–34. 6954864 10.1043/0003-3219(1982)052<0129:CILCWM>2.0.CO;2

[80] Subtelny J. A longitudinal study of soft tissue facial structures and their profile characteristics, defined in relation to underlying skeletal structures. American Journal of Orthodontics. 1959;45:481–507.

[81] Dahlbäck G. The Towns In: Helle K, editor. The Cambridge History of Scandinavia Prehistory to 1520. 1. Cambridge: Cambridge University Press; 2003 p. 611–34.

[82] Hager B, Foelsche U. Stable isotope composition of precipitation in Austria. Austrian Journal of Earth Sciences. 2015;108(2):2–13.

[83] Siegenthaler U, Oeschger H. Correlation of 18O in precipitation with temperature and altitude. Nature. 1980;285:314–7.

[84] Angel JL. Porotic hyperostosis. Anemias, malarias, and the marshes in the prehistoric eastern Mediterranean. Science. 1966(153):760–3. 5328679 10.1126/science.153.3737.760

[85] Angel JL. Porotic hypersotosis or osteoporosis symmetrica In: Brothwell D, Sandison AT, editors. Diseases in Antiquity. Springfield: Charles C Thomas; 1967 p. 373–89.

[86] Hrdlicka A. Anthropological work in Peru in 1913, with notes on on the pathology of the Ancient Peruvians. Washington: The Smithsoinian Institution; 1914.

[87] Moseley JE. The paleopathologic riddle of "symmetrical osteoporosis". The American Journal of Roentgenology Radium Therapy and Nuclear Medicine. 1965;95:135–42. 10.2214/ajr.95.1.135 14344352

[88] Roberts C, Manchester K. The Archaeology of Disease: Sutton Publishing Limited; 1995.

[89] Stuart-Macadam PL, Kent S, editors. Diet, Demography and Disease. New York: Aldine de Gruyter; 1992.

[90] Holck P. Two Medieval "Trepanations"—Therapy or Swindle? International Journal of Osteoarchaeology. 2008;18(2):188–94.

[91] Schaan DP. Is there a need to (un)gender the past? Advances in Gender Research. 2006;10:45–60.

[92] Schreiner KE. Om en trepanert finmarksskalle fra steinalderen. Oslo: Aschehoug; 1940.

[93] Torgersen J, Getz B, Simonsen P. Varanger-funnene I. Funn av menneskeskjeletter. Tromsø: Tromsø Museum; 1959.

[94] Jennbert K. Trepanations from the Stone Age to the Medieval Age in Scandinavian perspective In: Jennbert K, Larsson L, Petré R, Wyszomirska Werbart B, editors. Regions and Reflections In honour of Märta Strömberg. Acta Archaeologica Lund: Lund University: Department of Archaeology; 1991 p. 357–78.

[95] Bennike P. Ancient trepanations and differential diagnoses: A re-evaluation of skeletal remains from Denmark In: Arnott R, Finger S, Smith CUM, editors. Trepanation History-Discovery-Theory. Lisse: Swets & Zeitlinger; 2003 p. 95–115.

[96] Gross CG. Trepanation from the Palaeolithic to the internet In: Arnott R, Finger S, Smith CUM, editors. Trepanation History-Discovery-Theory. Lisse: Swets & Zeitlinger; 2003 p. 307–22.

[97] Martin G. Why trepan? Contributions from medical history and the South Pacific In: Arnott R, Finger S, Smith CUM, editors. Trepanation History-Discovery-Theory. Lisse: Swets & Zeitlinger; 2003 p. 323–45.

[98] Rocca J. Galen and the uses of trepanation In: ARNOTT R., FINGER S. & SMITH C. U. M (eds.) In: Arnott R, Finger S, Smith CUM, editors. Trepanation History-Discovery-Theory. Lisse: Swets & Zeitlinger; 2003 p. 253–71.

[99] Gensburg RS, Kawashima A, Sandler CM. Scintigraphic Demonstration of Lower Extremity Periostitis Secondary to Venous Insufficiency. Journal of Nuclear Medicine. 1988;29(7):1279–82. 3164753

[100] Nicholls SC. Sequelae of Untreated Venous Insufficiency. Seminars in Interventional Radiology. 2005;22(3):162–8. doi: 10.1055/s-2005-921960 21326689 10.1055/s-2005-921960 PMC3036289

